# Some of the Examination Questions

**Published:** 1919-08-02

**Authors:** 


### SISTER-TUTOR STUDENTSHIPS FOR COLLEGE NURSES.

### Some of the Examination Questions.

The test questions given at the examination for the sister-tutor studentships at King's College for Women on June 28 are full of interest, especially to those nurses who intend to become future candidates. The two papers comprised three questions on professional subjects and eighteen on subjects of general knowledge, wherein lay the great test of intelligence. Nine of those only were to be attempted, and the time allowed for both papers was three hours. In the professional paper the student had a splendid opportunity to excel, the questions being as follows:—

1. You are asked to give a lecture to second-year probationers on "The Pulse." Give the headings under which you would divide the lecture, and the special points to be described under each heading.

2. Describe the conditions resulting from complete fracture of the spine in the mid-dorsal region and the dangers which threaten the patient; discuss the chief points required in the nursing of such cases.

3. Write a short essay on one of the following subjects:—

(*a*) The advantages and disadvantages which have accrued to the nursing profession as a result of the 1914-1919 war.

(*b*) The special virtues which require cultivation in a nurse.

(*c*) The relation of a nurse to a sister, and that of a sister to a nurse.

As in the paper of the previous year, the questions on general knowledge ranged over a very wide area, yet were well within the scope of ordinary but necessarily up-to-date intelligence. A comparison of the two years' papers perhaps shows that the present one deals with more modern questions, and should thus be more easily answered. The topics include art, music, literature, geography, religion, and commerce. An account is required of the system of food rationing in this country, the object of town-planning schemes, and also that for which voluntary hospitals exist. The main reasons that the Coal Commission was appointed, and why the Panama Canal is of such commercial importance. What is meant by Disestablishment and Disendowment, and to give an account of any well-known religious controversy; also the names of ten living men and women who are sculptors, artists, architects, musicians, or authors. Who wrote "Middlemarch," "The Way of an Eagle," "Pilgrim's Progress," "The Elijah," "The Mikado," "Jane Eyre," "Martin Chuzzlewit," "Tannhauser," "Adventures of Sherlock Holmes," "The Bride of Lammermoor?" Where, and what are, the Kremlin, the Vatican, the Taj Mahal, Pyramids, Catacombs, the Acropolis, the Rubicon; and who are Clemenceau, Trotsky, Ludendorff, Haig, Alcock, Kerensky, Carson, Orlando, Northcliffe, Clynes? If going to Liverpool, Plymouth, Yarmouth, Sheffield, or Southampton, from which station in London would you start, and upon what railway company's lines would you travel; and in what country and upon what rivers are Petrograd, Belgrade, Strasburg, Quebec, Calcutta, Cairo, Bagdad, Cologne? Also to answer an advertisement requiring a matron for a school for 100 boys; to explain certain terms and abbreviations used in everyday language, and to show why the recent discovery of oil in Derbyshire should arouse such interest. Finally, the provisions of any Act of Parliament passed within the last two years, or of any Bill now before Parliament. Having answered these questions and achieved success, a nurse may well be proud of her prowess.

